# Prostate Artery Embolization: Challenges, Tips, Tricks, and Perspectives

**DOI:** 10.3390/jpm13010087

**Published:** 2022-12-29

**Authors:** Benjamin Moulin, Massimiliano Di Primio, Olivier Vignaux, Jean Luc Sarrazin, Georgios Angelopoulos, Antoine Hakime

**Affiliations:** Diagnostic and Interventional Radiology, American Hospital of Paris, 92200 Neuilly sur Seine, France

**Keywords:** prostate adenoma, benign prostate hypertrophia, embolization, prostate artery embolization, minigù mally invasive

## Abstract

Prostatic artery embolization (PAE) consists of blocking the arteries supplying the prostate to treat benign prostate hypertrophia (BPH). Its effectiveness on both urinary symptoms and flowmetric parameters has now been amply demonstrated by around a hundred studies, including several randomized trials. The main advantage of this procedure is the very low rate of urinary and sexual sequelae, including ejaculatory, with an excellent tolerance profile. The arterial anatomy is a key element for the realization of PAE. Its knowledge makes it possible to anticipate obstacles and prevent potential complications related to nontarget embolization. Nontarget embolization can occur with a small intraprostatic shunt or reflux and has no consequences except some local inflammation symptoms that resolve in a couple of days. Nevertheless, some situations with large arterial shunts arising from the prostatic artery must be recognized (accessory rectal, bladder, or pudendal branches), and must imperatively be protected before embolization, at the risk of exposing oneself to otherwise ischemic complications that are more severe, such as bladder necrosis and skin or mucosal necrosis. This article offers a step-by-step review of the various anatomical and technical key points to ensure technical and clinical success, while avoiding the occurrence of adverse events.

## 1. Introduction

A lower urinary tract symptom (LUTS) is a very common condition with significant socio-economic importance to the public health system. Benign prostatic hyperplasia (BPH) is the first etiology of a LUTS in men, with an incidence of 50% among men in the fifth decade [1]. The treatment is usually based on a conservative approach, pharmacological therapy, and surgical procedures. Trans-urethral resection of the prostate (TURP) is the standard therapy for the management of benign prostate enlargement (BPE), after the failure of medical therapy. Nevertheless, TURP is associated with relevant morbidity, including retrograde ejaculation and incontinence (early or permanent), and may present an adverse event such as infection, bleeding, and urethral stricture [2,3]. In this context, minimally invasive therapeutics emerged in the last decade, trying to ensure patients lower adverse event rates. Among them, prostate artery embolization (PAE) was first described in 2000 and consists of endovascular embolization of the prostate arteries with small, calibrated particles, which leads to an interruption of blood supply and then to prostate tissue necrosis and atrophy. This procedure offers advantages compared to surgery, such as the possibility to perform embolization under local anesthesia, without a Foley catheter or endourethral instrument insertion [4]. Additionally, PAE pretends to preserve sexual and urinary function in almost all cases, which seems essential given the expectations of patients [5]. On another hand, PAE remains less studied than TURP or other surgical technics, such as laser ablation. The purpose of this paper is to review the technical aspect and current perspective of PAE. Based on the experience and literature review, the authors provided a step-by-step approach with the first part of the manuscript focusing on technical aspects and challenges during embolization, and the second part reviewing the current evidence and controversies of this procedure.

## 2. Tips and Tricks to Perform Prostate Artery Embolization (PAE)

### 2.1. Perform an Arterial Mapping: Preoperative Computerized Tomography (CT) Angiogram or Intraoperative Cone-Beam CT (CBCT)

Cross-sectional imaging with vascular opacification is necessary before embolization for good anatomical understanding, the search for pitfalls, and the planning of the intervention. This imaging can be a CT angiogram performed prior to intervention (at the time of the pre-intervention assessment) or a CBCT angiogram performed at the beginning of the procedure. There are no clear guidelines on this topic. We suggest favoring the CT, given that it enables the easiest approach (radial or femoral) to be chosen before the intervention, and that it avoids injection of iodinated contrast media at the time of the procedure. Indeed, the accumulation of contrast in the bladder can prove to be problematic due to the discomfort encountered and the risk of urinary retention (teams using CBCT can have recourse to the placement of a penis sheath, or more rarely, an indwelling catheter, which from our point of view, is too invasive and thus we are in favor of performing arterial mapping by a pre-operative CT angiogram).

### 2.2. Manage Aortoiliac Anatomy

Aortoiliac imaging analysis is important for choosing the easier approach. In classical situations, radial or femoral roads can be performed without differences, depending on operator experience and preferences. For atheromatous patients, which commonly present with very sinuous vessels, aortoiliac catheterism can be very complicated in particular cross-over maneuvers or ipsilateral iliac catheterization. Similarly, an important iliac bifurcation angulation can be conducted to a difficult homo-lateral internal iliac catheterization and can request a second ipsilateral femoral puncture.

**Figure 1:**
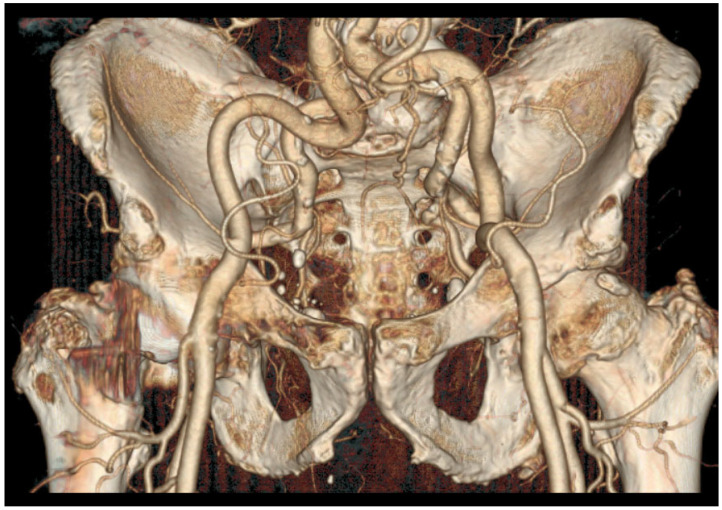
A CT scan with a 3D reconstruction showing very tortuous iliac arteries and very acute right internal/external iliac angulation. Upper radial access was privileged in the first intention.

A radial approach should be considered in the following situations:

- Tortuous common iliac arteries (Figure 1);
- Aortic or iliac bifurcation forming a very acute angle.

### 2.3. Identify the Origin of the Prostatic Artery

**Figure 2:**
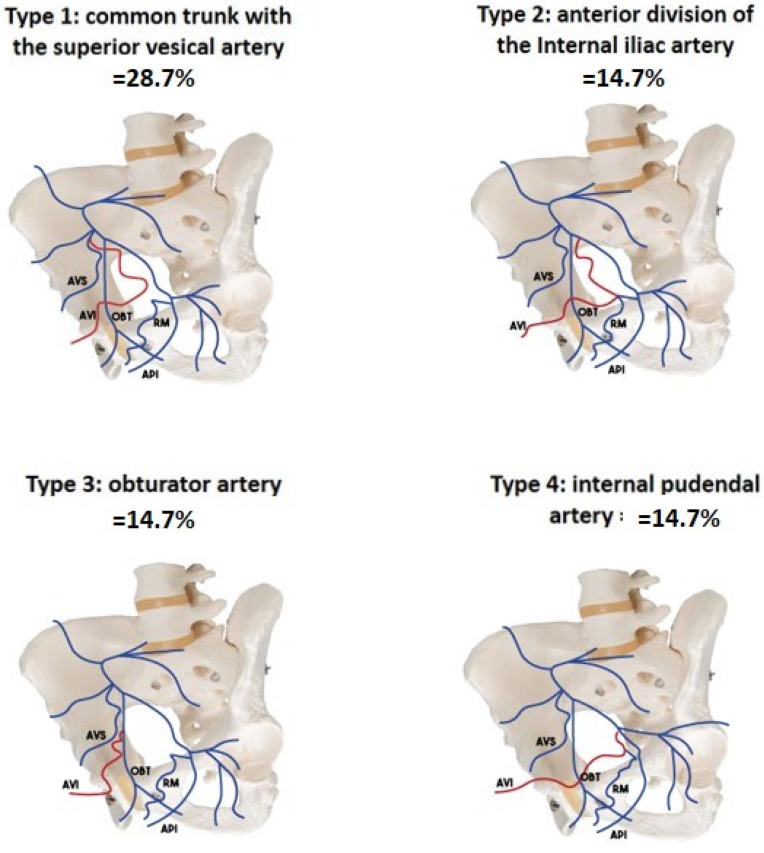
Prostatic artery origin description, from de Assis [6].

Many studies have been published on the anatomy of the prostatic arteries. The most used classification is the one described by De Assis et al. (Figure 2), which classifies the anatomy into five types, depending on the origin of the prostatic artery (type I, II, III, and IV). Type V (5.6%) is a combination of situations that do not correspond to types 1 to 4 and correspond to prostatic arteries arising from a trifurcation of the anterior trunk of the internal iliac artery, gluteal artery, accessory pudendal artery, or epigastric artery.

**Figure 3:**
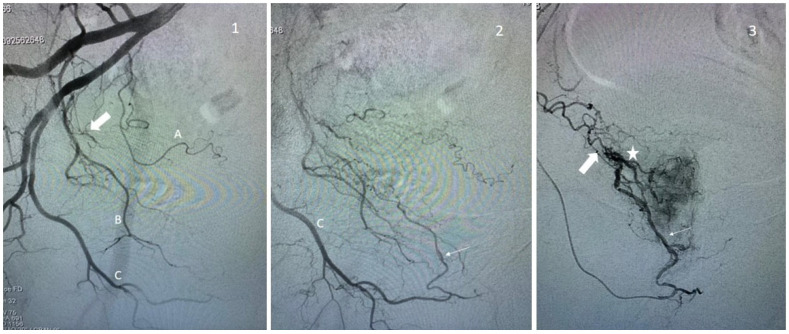
Examples of prostate artery occlusion and revascularization of the prostate via a distal pudendal/prostatic apical shunt. **Image 1**: Internal iliac angiography demonstrating a vesical inferior artery (A), an obturator artery (B), and an internal pudendal artery (C). Note the occluded prostatic artery (arrow). **Image 2**: Supraselective angiography of the internal pudendal artery (C) showing a connexion with the prostatic artery via an apical shunt (thin arrow). **Image 3**: Retrograde catheterism of the prostatic artery (thin arrow) and opacification demonstrating opacification of the prostatic central branch (star) and confirming occlusion of a proximal prostatic artery (large arrow).

When no prostatic artery is found in the territory of the internal iliac, one should always consider looking for a possible origin from the external iliac via an accessory obturator artery (1.8% of patients [7]) or via the epigastric artery (the so-called “corona mortis artery”). Additionally, in the cases of occlusion of the prostatic artery and, in particular, of the central artery, the flow is usually supplied by collaterality, by a frequency in the distality of the internal pudendal artery (Figure 3), the peripheral or contralateral prostatic artery, and the obturator artery.

### 2.4. Manage the Prostatic Artery Catheterism

For an operator accustomed to endovascular navigation, catheterization of the prostatic artery is usually easy, once its origin has been identified. However, certain situations can make this catheterism very delicate. The risk factors making this catheterization potentially difficult have previously been reported [8]:

- Atheroma and sinuous arteries (increasing with age);
- Type 1 prostatic artery, with tight angulation between the anterior trunk of the internal iliac and the inferior bladder artery (this situation also sometimes occurs with the internal pudendal artery on type 4).

Here are some examples, as well as some tips to remedy them.

#### 2.4.1. When the Internal Iliac Arteries Are Tortuous or When the Catheter Is Unstable 

This situation can be solved

- with the use of a longer sheath (45 centimeters for example), when a femoral approach is intended;
- with the “buddy-wire technic” [9]: the use of an increased sheath caliber (7 french) and the positioning of a guidewire parallel to the catheter, to increase the stability of this one;
- and with a radial approach [10], which can be considered to overcome the loss of stability inherent in the cross-over.

#### 2.4.2. When the Prostatic Artery Arises with a Very Acute Angulation 

Pre-formed micro-catheters with significant terminal angulation can be used [9] (terminal angulation can also be personally shaped with steam). Torqueable microcatheters are also available [11]. The end of the microguide can also be modified (double curvature) to allow the strong angulation to pass. Torque microcatheters are also available. Additionally, the extremity of the microguide can also be shaped with a double curve.

- Use a rigid torque catheter with a tight distal curve to directly catheterize the artery without a microcatheter. This type of catheter should be handled with care, as it is very rigid and can easily lead to dissection of the prostatic artery.

### 2.5. Know the Intra-Prostatic Anatomy, Detect and Protect the Shunt and Collaterals

**Figure 4:**
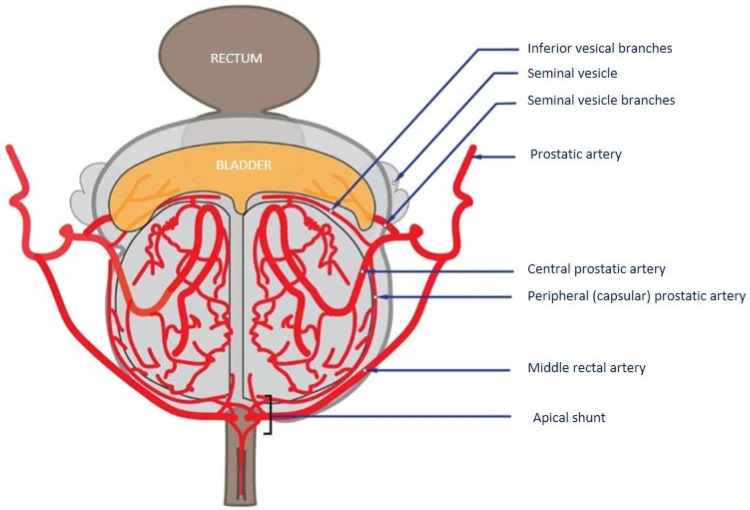
Vascular intra-prostatic anatomy (initial description by Picel) [12].

The prostatic artery is divided into two branches (Figure 4), the superior or anteromedial artery which supplies the central prostate (often called the central artery for convenience), and the inferior or posteromedial artery, which supplies the peripheral prostate (also called the capsular or peripheral artery). In about 8% of cases [5], the two branches of the prostatic artery have an independent birth. It is then necessary to specifically identify the central branch which will be the target of the embolization.

The origin of the inferior vesical, vesicular, and middle rectal branches, when present, is generally upstream of the central/peripheral prostatic artery bifurcation. A lot of connections have been described between the prostatic artery and other pelvic arteries. The most frequent are collateral with inferior vesical, middle rectal, and internal pudendal arteries.

#### 2.5.1. Middle Rectal and Inferior Vesical Collaterals

**Figure 5:**
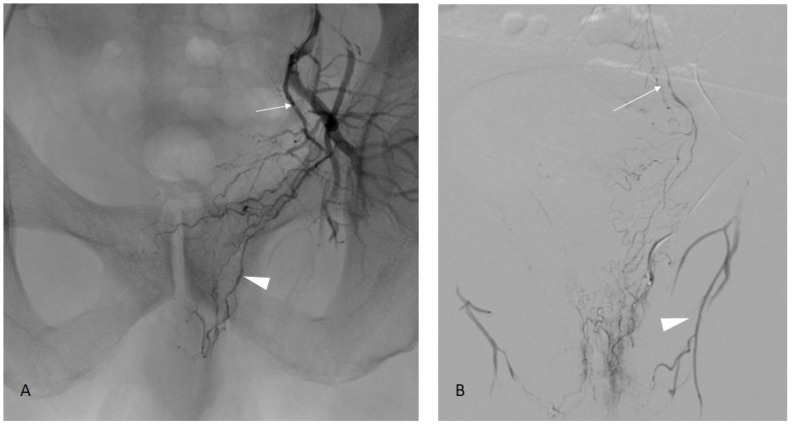
Prostate artery connection with the middle rectal artery. Image (**A**): Iliac angiography demonstrating a vesico-prostatic trunk (arrow) with collateral in the direction of the rectum (arrowhead). Image (**B**): Supraselective angiography in the middle rectal artery confirming the opacification of the rectum and connection with the superior rectal artery (arrow) arising from the inferior mesenteric artery. Note another shunt with the obturator artery (arrowhead).

Collateral to the rectum (Figure 5, middle rectal branch, 14%) or bladder (inferior bladder arteries, 11%) are frequent situations [13]. If the prostatic branches can be catheterized supraselectively, these can be embolized directly. If supraselective embolization is not feasible, the middle rectal or lower bladder branch must be occluded using a microcoil, which redirects the flow to the prostatic artery.

#### 2.5.2. Internal Pudendal Artery Collateral

Collaterals are frequently found with the internal pudendal arteries. These are clearly visible for a trained operator on supraselective DSA performed in the prostatic arteries, but can also be seen on preoperative or CBCT scans when the prostatic arteries are enhanced.

**Figure 6:**
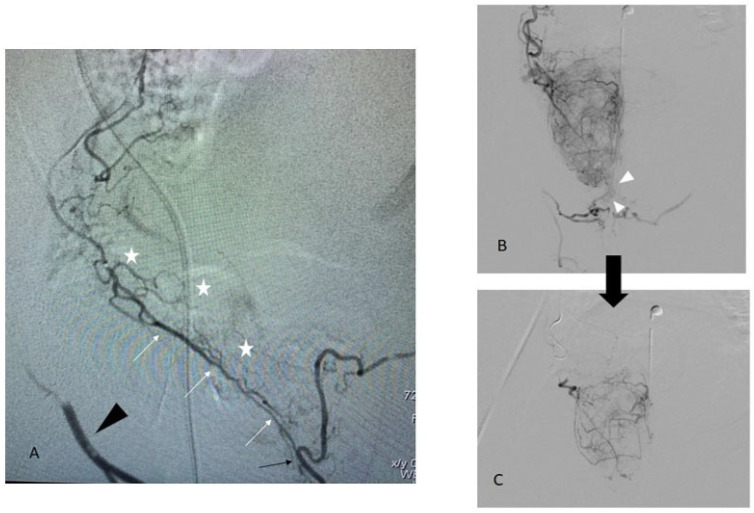
Different patterns of a shunt between prostatic and penile arteries. Image (**A**): an accessory pudendal artery arising from the peripheral prostatic artery (white arrow) with a direct connection to the internal pudendal artery (black arrow) which is retrogradely opacified (black arrowhead). Prostate vascularization (white stars) arises from small branches along the aPA. In this case, embolization should not be performed without protection of the anastomosis [14]. Image (**B**): an intra-prostatic capsular shunt (arrowhead) with a high injection rate (0.7 cc/s). Unlike image A, there is no real individualizable branch between prostatic and penile territory. Image (**C**): at a lower injection rate (0.3 cc/s), the shunt is no longer present. Embolization can be performed safely.

Two situations should be distinguished (Figure 6):

- **The post-capsular collaterals**, which are usually found at the apex, are opacified during DSA with a flow rate usually superior to 0.5 cc/s. These collaterals disappear with the decrease in the injection rate. In these cases, embolization can be performed without risk but must be performed at a low flow rate;
- **The pre-capsular collaterals**, which are true intra-prostatic arterial connections (usually described as the “accessory pudendal artery” (aPA)). In some extreme situations, there is no real prostatic artery, and the prostate is vascularized by several small branches along the latero-prostatic course of the aPA, which then gives the penile vascularization. As soon as the connections with the internal pudendal artery are of a certain size (we can consider that the visibility of a true course in angiography is a good cut-off) there is a significant risk of non-target embolization, including with low-flow embolization. These connections must, therefore, be protected prior to embolization.

**How to manage a large connection from the prostatic to the internal pudendal artery?**

- First case: the central prostatic branches can be supraselectively catheterized. In this case, a very careful embolization can be performed. Most attention must be paid to avoid reflux into the aPA.
- Second case: a supraselective catheterization is impossible. It is then necessary to occlude the accessory pudendal artery in its post-prostatic portion to be able to embolize upstream “by collaterality” of the prostatic branches. Nevertheless, the consequences of the occlusion of the aPA on penile vascularization must be taken into account. A recent study found no difference between a patient who received embolization of penile collateral in terms of erectile function [14]. These are still debated, despite numerous studies on the subject in the context of radical prostate surgeries for prostate cancer. These anatomical studies classified penile vascularization according to three categories [15] depending on whether the vascularization is performed only by the internal pudendal (type I, 61.9%), by the internal and accessory pudendal (type II, 32.8%), or only via the accessory pudendal arteries (type III, 5.4%). Put another way, this means that when an aPA is founded, it is the only supply to the penile in 14% of the case (type III/(type II + III)). By cross-referencing these data with MacLean [14], who reported around 12% of penile/aPA protection coiling during PAE, we arrived at a number of 1.7% (12% × 14%) of patients who are potentially at risk of protective occlusion of a type III vascularization. We, therefore, recommend before occluding an aPA to ensure that it is not the only artery supplying the penis, in which case the risk of post-occlusion impotence seems real to us. In our experience, the presence of selectively non-catheterizable prostatic branches in the context of type III penile vascularization in young subjects wishing to preserve their sexual activity is the only situation contraindicating embolization.

Ultimately, the study of the prostate and penile anatomy and the possible interconnections between these two systems seems to be an essential time in the preoperative analysis.

From our point of view, this is an additional argument in favor of the use of preoperative CT. Indeed, when the CT scan is carried out according to an adequate protocol, connections of significant size can be detected easily, and give the operator the possibility to anticipate at-risk situations and adapt the procedure to the anatomy.

## 3. Perspective, Evolution, and Discussion about PAE

### 3.1. PAE Safety and Complications

The safety of PAE procedures from the prospective study is presented in Table 1, including four randomized clinical trials (RCT) of PAE vs. TURP, 1 RCT of PAE vs. an open adenomectomy, one RCT of PAE vs. a sham procedure, and one prospective cohort on PAE only. The results show an excellent safety profile with an absence of grade 4 and 5 adverse events, and grade 3 adverse events in only one study, affecting 4.3% of the total patients treated with PAE. Grade I adverse events range from 7.5 to 67.3% of patients and grade II from 1 to 47.8%. The significant variability between studies is probably explained by the fact that some studies have included the symptoms of post-embolization syndrome in the complications whereas others did not. These data confirm that PAE is a safe procedure, with a few side effects except the post-embolization syndrome.

### 3.2. PAE Limitations

Results of the RCT conducted by Abt demonstrated no difference in IPSS score improvement between PAE and TURP. Nevertheless, TURP seems to achieve better results in terms of debimetric results [23]. To date, no large prospective study with a long follow-up has been published to compare these two technics. PAE should have a higher recurrence rate, as a recent publication reported a reintervention rate of 58% at 10 years [24], whereas TURP seems to have a reintervention rate of around 20% [3].

### 3.3. Embolization Agent

Historically, EAP was performed with particles. Recently some studies have been published with the use of alternative embolic agents such as n-butyl-cyanoacrylate, ethylene vinyl alcohol copolymer, and alcohol [25,26]. A recent study [27] showed a decrease in fluoroscopy time and radiation dose with the use of glue, without a difference in terms of IPSS score.

### 3.4. Particle Size

The size of the ideal particles to be used remains subject to discussion. A randomized trial [28] comparing the use of 100–300 vs. 300–500 micron caliber particles did not show a significant difference between the two groups. Nevertheless, various studies have shown a more extensive prostatic necrosis on post-operative MRI, the greatest decrease in prostatic volume and PSA level was when the size of the particles decrease [28,29]. It would make sense that a more aggressive embolization would allow greater prostatic necrosis and, therefore, a greater clinical benefit, in particular on the risk of recurrence in the medium/long term. However, this remains to be proven, and the aggressiveness of the embolization must be weighed against the undoubtedly increased risk of nontarget embolization, as the small particles cross the small shunts more easily. Our team tends to favor aggressive embolization (a particle caliber from 50 to 300 microns) when supra-selective angiography of the prostatic artery does not show that any collateral potentiality is at risk of nontarget embolization, but these remain out of the guidelines which recommend to use 300–500 micron particles [4].

### 3.5. Peripheral vs. Central Prostate

The prostate adenoma develops mainly at the expense of the transition zone. Considering both the fact that the adenoma is vascularized almost exclusively by the central branch of the prostatic artery and that, on the contrary, the majority of extra-prostatic anastomoses arise from the peripheral artery (78% of cases [30]), the idea of an aggressive embolization of the central artery (particles of calibers less than 150 microns or embolization with a liquid agent) could be attractive, thus supplementing it with micro-particles of a higher caliber for the continuation of the embolization. Once again, studies would be needed to find out if such an attitude offers better results, and current guidelines do not recommend it.

In conclusion, PAE is a technically and intellectually challenging intervention. A precise knowledge of both theoretical and angiographic anatomy is essential in order to embolize the entire adenoma while avoiding nontarget embolization. The learning curve is arguably one of the longest of all vascular radiology procedures, but performed by a trained operator, both technical and clinical success rates are excellent, with a low risk of major complication. 

## Figures and Tables

**Table 1:** Adverse events after PAE from seven prospective studies [16,17,18,19,20,21,22].

Study	Pisco, 2019 [19]	Insauti, 2020 [18]	Abt, 2018 [16]	Carnevale, 2015 [17]	Russo, 2015 [21]	Ray, 2018 [20]	Salem, 2018 [22]
Patient Number	n = 78	n = 23	n = 48	n = 15	n = 80	n = 199	n = 45
Design	RCT, PAE vs. Sham	RCT, PAE vs. TURP	RCT, PAE vs. TURP	RCT, PAE vs. TURP	RCT, PAE vs. prostatectomy	Registry-based study	Prospective, PAE only
**Total adverse event, n (%)**	25 (32.0)	15 (65.2)	36 (75)	4 (46.7)	7 (8.7)	136 (68.3)	26 (57.8)
*Clavien Dindo grade*							
grade I	21 (26.9)	4 (17.3)	54%		6 (7.5)	134 (67.3)	24 (53.3)
grade II	3 (3.8)	11 (47.8)	17%		1 (1.3)	2 (1)	2 (4.4)
grade III	1 (1.3)	0	4.30%		0		0
grade IV	0	0			0		0
grade V	0	0			0		0
*Description (number, %)*							
Urinary frequency and urgency							3 (6.6)
Burning perineal pain	1 (1.3)	1 (4.3)	15 (31.3)				
Burning urethral pain	3 (3.8)	4 (17.3)					
Dysuria	3 (3.8)				5 (6.3)		13 (28.9)
Ecchymosis	2 (2.6)						
Haematospermia	7 (9.0)			1 (6.7)	1 (1.3)	25 (12.6)	2 (4.4)
Haematuria	5 (6.4)	1 (4.3)	4 (8.3)	2 (13.3)		37 (18.6)	6 (13.3)
Inguinal haematoma	4 (5.1)					4 (2)	
Penile ulcer						2 (1)	
Artery dissection						4 (2.0)	
Acute urinary retention		5 (21.7)	1 (2.1)				2 (4.4)
Radiodermitis		1 (4.3)					
Erectile dysfunction		1 (4.3)					
Change in ejaculation volume		1 (4.3)		2 (13.3)		48 (24.1)	
Incontinence						2 (1)	
Prostate fragment expelled	1 (1.3)						
Rectorrhagia/rectal ischemia	2 (2.6)	1 (4.3)		1 (6.7)			
Urinary tract infection	1 (1.3)		10 (20.1)		1 (1.3)	14 (7.0)	
Other			6 (12.5)	1 (6.7)			

## Data Availability

Not applicable.

## References

[1] Berry S.J., Coffey D.S., Walsh P.C., Ewing L.L. (1984). The Development of Human Benign Prostatic Hyperplasia with Age. J. Urol..

[2] Ahyai S.A., Gilling P., Kaplan S.A., Kuntz R.M., Madersbacher S., Montorsi F., Speakman M.J., Stief C.G. (2010). Meta-analysis of Functional Outcomes and Complications Following Transurethral Procedures for Lower Urinary Tract Symptoms Resulting from Benign Prostatic Enlargement. Eur. Urol..

[3] Reich O., Gratzke C., Bachmann A., Seitz M., Schlenker B., Hermanek P., Lack N., Stief C.G. (2008). Urology Section of the Bavarian Working Group for Quality Assurance† Morbidity, Mortality and Early Outcome of Transurethral Resection of the Prostate: A Prospective Multicenter Evaluation of 10,654 Patients. J. Urol..

[4] Cornelis F.H., Bilhim T., Hacking N., Sapoval M., Tapping C.R., Carnevale F.C. (2020). CIRSE Standards of Practice on Prostatic Artery Embolisation. Cardiovasc. Intervent. Radiol..

[5] Malde S., Umbach R., Wheeler J.R., Lytvyn L., Cornu J.-N., Gacci M., Gratzke C., Herrmann T.R.W., Mamoulakis C., Rieken M. (2021). A Systematic Review of Patients’ Values, Preferences, and Expectations for the Diagnosis and Treatment of Male Lower Urinary Tract Symptoms. Eur. Urol..

[6] de Assis A.M., Moreira A.M., de Paula Rodrigues V.C., Harward S.H., Antunes A.A., Srougi M., Carnevale F.C. (2015). Pelvic Arterial Anatomy Relevant to Prostatic Artery Embolisation and Proposal for Angiographic Classification. Cardiovasc. Intervent. Radiol..

[7] Bilhim T., Pisco J., Pinheiro L.C., Rio Tinto H., Fernandes L., Pereira J.A. (2014). The Role of Accessory Obturator Arteries in Prostatic Arterial Embolization. J. Vasc. Interv. Radiol..

[8] du Pisanie J., Abumoussa A., Donovan K., Stewart J., Bagla S., Isaacson A. (2019). Predictors of Prostatic Artery Embolization Technical Outcomes: Patient and Procedural Factors. J. Vasc. Interv. Radiol..

[9] Bagla S., Isaacson A.J. (2016). Tips and Tricks for Difficult Prostatic Artery Embolization. Semin. Interv. Radiol..

[10] Bhatia S., Harward S.H., Sinha V.K., Narayanan G. (2017). Prostate Artery Embolization via Transradial or Transulnar versus Transfemoral Arterial Access: Technical Results. J. Vasc. Interv. Radiol..

[11] Dudeck O. (2014). Safety and Efficacy of Target Vessel Catheterization with the New Steerable Microcatheter Direxion Compared with a Standard Microcatheter: A Prospective, Preclinical Trial. Cardiovasc. Intervent. Radiol..

[12] Picel A.C., Hsieh T.-C., Shapiro R.M., Vezeridis A.M., Isaacson A.J. (2019). Prostatic Artery Embolization for Benign Prostatic Hyperplasia: Patient Evaluation, Anatomy, and Technique for Successful Treatment. RadioGraphics.

[13] Bilhim T., Pisco J.M., Rio Tinto H., Fernandes L., Pinheiro L.C., Furtado A., Casal D., Duarte M., Pereira J., Oliveira A.G. (2012). Prostatic Arterial Supply: Anatomic and Imaging Findings Relevant for Selective Arterial Embolization. J. Vasc. Interv. Radiol..

[14] Maclean D., Vigneswaran G., Maher B., Hadi M., Harding J., Harris M., Bryant T., Hacking N., Modi S. (2022). The effect of protective coil embolization of penile anastomoses during prostatic artery embolization on erectile function: A propensity-matched analysis. J. Vasc. Interv. Radiol..

[15] Henry B.M., Pękala P.A., Vikse J., Sanna B., Skinningsrud B., Saganiak K., Walocha J.A., Tomaszewski K.A. (2017). Variations in the Arterial Blood Supply to the Penis and the Accessory Pudendal Artery: A Meta-Analysis and Review of Implications in Radical Prostatectomy. J. Urol..

[16] Abt D., Hechelhammer L., Müllhaupt G., Markart S., Güsewell S., Kessler T.M., Schmid H.-P., Engeler D.S., Mordasini L. (2018). Comparison of prostatic artery embolisation (PAE) versus transurethral resection of the prostate (TURP) for benign prostatic hyperplasia: Randomised, open label, non-inferiority trial. BMJ.

[17] Carnevale F.C., Iscaife A., Yoshinaga E.M., Moreira A.M., Antunes A.A., Srougi M. (2016). Transurethral Resection of the Prostate (TURP) Versus Original and PErFecTED Prostate Artery Embolization (PAE) Due to Benign Prostatic Hyperplasia (BPH): Preliminary Results of a Single Center, Prospective, Urodynamic-Controlled Analysis. Cardiovasc. Intervent. Radiol..

[18] Insausti I., Sáez de Ocáriz A., Galbete A., Capdevila F., Solchaga S., Giral P., Bilhim T., Isaacson A., Urtasun F., Napal S. (2020). Randomized Comparison of Prostatic Artery Embolization versus Transurethral Resection of the Prostate for Treatment of Benign Prostatic Hyperplasia. J. Vasc. Interv. Radiol..

[19] Pisco J.M., Bilhim T., Costa N.V., Torres D., Pisco J., Pinheiro L.C., Oliveira A.G. (2020). Randomised Clinical Trial of Prostatic Artery Embolisation Versus a Sham Procedure for Benign Prostatic Hyperplasia. Eur. Urol..

[20] Ray A.F., Powell J., Speakman M.J., Longford N.T., DasGupta R., Bryant T., Modi S., Dyer J., Harris M., Carolan-Rees G. (2018). Efficacy and safety of prostate artery embolization for benign prostatic hyperplasia: An observational study and propensity-matched comparison with transurethral resection of the prostate (the UK-ROPE study). BJU Int..

[21] Russo G.I., Kurbatov D., Sansalone S., Lepetukhin A., Dubsky S., Sitkin I., Salamone C., Fiorino L., Rozhivanov R., Cimino S. (2015). Prostatic Arterial Embolization vs Open Prostatectomy: A 1-Year Matched-pair Analysis of Functional Outcomes and Morbidities. Urology.

[22] Salem R., Hairston J., Hohlastos E., Riaz A., Kallini J., Gabr A., Ali R., Jenkins K., Karp J., Desai K. (2018). Prostate Artery Embolization for Lower Urinary Tract Symptoms Secondary to Benign Prostatic Hyperplasia: Results From a Prospective FDA-Approved Investigational Device Exemption Study. Urology.

[23] Knight G.M., Talwar A., Salem R., Mouli S. (2021). Systematic Review and Meta-analysis Comparing Prostatic Artery Embolization to Gold-Standard Transurethral Resection of the Prostate for Benign Prostatic Hyperplasia. Cardiovasc. Intervent. Radiol..

[24] Bilhim T., Costa N.V., Torres D., Pinheiro L.C., Spaepen E. (2022). Long-Term Outcome of Prostatic Artery Embolization for Patients with Benign Prostatic Hyperplasia: Single-Centre Retrospective Study in 1072 Patients Over a 10-Year Period. Cardiovasc. Intervent. Radiol..

[25] Moulin B., Hakime A., Kuoch V. (2022). Percutaneous Prostatic Artery Embolization with Absolute Alcohol: A Case Report. J. Vasc. Interv. Radiol..

[26] Chau Y., Rambaud-Collet C., Durand M., Léna P., Raffaelli C., Brunner P., Quintens H., Sédat J. (2018). Prostatic Artery Embolization with Ethylene Vinyl Alcohol Copolymer: A 3-Patient Series. J. Vasc. Interv. Radiol..

[27] Salet E., Crombé A., Grenier N., Marcelin C., Lebras Y., Jambon E., Coussy A., Cornelis F.H., Petitpierre F. (2022). Prostatic Artery Embolization for Benign Prostatic Obstruction: Single-Centre Retrospective Study Comparing Microspheres Versus n-Butyl Cyanoacrylate. Cardiovasc. Intervent. Radiol..

[28] Torres D., Costa N.V., Pisco J., Pinheiro L.C., Oliveira A.G., Bilhim T. (2019). Prostatic Artery Embolization for Benign Prostatic Hyperplasia: Prospective Randomized Trial of 100–300 μm versus 300–500 μm versus 100- to 300-μm + 300- to 500-μm Embospheres. J. Vasc. Interv. Radiol..

[29] Wang M.Q., Zhang J.L., Xin H.N., Yuan K., Yan J., Wang Y., Zhang G.D., Fu J.X. (2018). Comparison of Clinical Outcomes of Prostatic Artery Embolization with 50-μm Plus 100-μm Polyvinyl Alcohol (PVA) Particles versus 100-μm PVA Particles Alone: A Prospective Randomized Trial. J. Vasc. Interv. Radiol..

[30] Anract J., Amouyal G., Peyromaure M., Zerbib M., Sapoval M., Barry Delongchamps N. (2019). Study of the intra-prostatic arterial anatomy and implications for arterial embolization of benign prostatic hyperplasia. Prog. Urol..

